# Klatskin Tumor in the Light of ICD-O-3: A Population-Based Clinical Outcome Study Involving 1,144 Patients from the Surveillance, Epidemiology, and End Result (SEER) Database (2001-2012)

**DOI:** 10.7759/cureus.18941

**Published:** 2021-10-21

**Authors:** Jaffar Khan, Asad Ullah, Nathaniel Matolo, Abdul Waheed, Noor Nama, Tahir Khan, Bisma Tareen, Zarmina Khan, Sohni G Singh, Frederick D Cason

**Affiliations:** 1 Pathology and Laboratory Medicine, Indiana University School of Medicine, Indianapolis, USA; 2 Pathology, Medical College of Georgia - Augusta University, Augusta, USA; 3 Surgical Oncology, San Jaoquin General Hospital, Jaoquin, USA; 4 Surgery, San Jaoquin General Hospital, San Jaoquin, USA; 5 Obstetrics and Gynaecology, Bolan Medical College Complex Hospital, Quetta, PAK; 6 Internal Medicine, Sandman Provincial Hospital, Quetta, PAK; 7 Internal Medicine, Bolan Medical College, Quetta, PAK; 8 Obstetrics and Gynaecology, Sandman Provincial Hospital, Quetta, PAK; 9 Surgery, San Joaquin General Hospital, San Joaquin, USA

**Keywords:** extrahepatic cholangiocarcinoma, intrahepatic cholangiocarcinoma, seventh decade, surgical resection, lethal biliary malignancy, international classification of diseases for oncology, cholangiocarcinoma, klatskin tumor

## Abstract

Introduction

Klatskin tumors (KTs) occur at the confluence of the right and left extrahepatic ducts and are classified based on their anatomical and histological codes in the International Classification of Diseases for Oncology (ICD-O). The second edition of the ICD-O (ICD-O-2) allocated a distinctive histological code to KT, which also included intrahepatic cholangiocarcinoma (CC). This unclear coding may result in ambiguous reporting of the demographic and clinical features of KT. The current study aimed to investigate the demographic, clinical, and pathological factors affecting the prognosis and survival of KT in the light of the updated third edition of ICD-O, Ninth Revision (ICD-O-3).

Methods

Data of 1,144 patients with KT from the Surveillance, Epidemiology, and End Result (SEER) database (2001-2012) were extracted. Patients with KT were analyzed for age, sex, race, stage, treatment, and long-term survival. The data were analyzed using chi-square tests, t-tests, and univariate and multivariate analyses. The Kaplan-Meier analysis was used to compare long-term survival between KT and subgroups of all biliary CCs.

Results

Of all biliary CCs, KT comprised 9.35%, with a mean age of diagnosis of 73±13 years, and was more common in men (54.8%) and Caucasian patients (69.5%). Histologically, moderately differentiated tumors were the most common (38.9%) followed by poorly differentiated (35.7%), well-differentiated (23.3%), and undifferentiated tumors (2.2%) (p<0.001). Most tumors in the KT group were 2-4 cm in size (41.5%), while fewer were >4 cm (29.7%) and <2 cm (28.8%) (*p<*0.001). ICD-O-3 defined most KTs in extrahepatic location (53.5%), while the remainder were in other biliary locations (46.5%) (p<0.001). Most KT patients received no treatment (73%), and for those who were treated, the most frequent modality was radiation (52.7%), followed by surgery (28.1%), and both surgery and radiation (19.2%) (*p<*0.001). Mean survival time for KT patients treated with surgery was inferior to all CCs of the biliary tree (1.72±2.61 vs. 1.87±2.18 years) (*p=*0.047). Multivariate analysis identified regional metastasis (OR=2.8; 95% CI=2.6-3.0), distant metastasis (OR=2.1; 95% CI=1.9-2.4), lymph node positivity (OR=1.6; 95% CI=1.4-1.8), Caucasian race (OR=2.0; 95% CI=1.8-2.2), and male sex (OR=1.2; 95% CI=1.1-1.3) were independently associated with increased mortality for KT (p<0.001).

Conclusion

The ICD-O-3 has permitted a greater understanding of KT. KT is a rare and lethal biliary malignancy that presents most often in Caucasian men in their seventh decade of life with moderately differentiated histology. Surgical resection does not provide any survival advantage compared to similarly treated biliary CCs. In addition, the combination of surgery and radiation appeared to provide no added survival benefits compared to other treatment modalities for KT.

## Introduction

Klatskin tumors (KTs), also known as hilar cholangiocarcinomas (CCs) or Altemeier-Klatskin tumors, first described in late 1965 and named after Dr. Gerald Klatskin, are a rare entity of extrahepatic CCs arising at the confluence of the right and left hepatic ducts [1,2]. It is the most common type of CCs, accounting for approximately 60-80% of all CCs reported each year in the United States [3]. KT also accounts for approximately 2% of all cancer diagnoses, with an overall incidence of 2-4 cases/100,000 population/year patients and is seen slightly more frequently in males (male:female ratio of 1.3:1) [4]. Almost two-thirds of KT cases occur in patients over the age of 65 years, with a near 10% increase in patients over 80 years of age [5].

KTs are classified by the International Classification of Diseases for Oncology (ICD-O) based on their unique anatomical code (topographical code) and histological code (morphological code) in the Surveillance, Epidemiology, and End Result (SEER) database [6]. In version 1 of ICD-O (ICD-O-1), KTs were not assigned a unique histological and topographical code, and they were reported as either intrahepatic or extrahepatic CC [6]. In version 2 of the ICD-O (ICD-O-2), the unique histological code of KT is included in the topographical code of intrahepatic CC, resulting in a considerable error in reporting KT [6].

To examine the impact of this misclassification on site-specific CCs, Welzel et al. calculated the annual percentage changes from the SEER database using the ICD-O-2 classification. They found that 269 KT were found between 1992 and 2000 using ICD-O-2 from the SEER database; 91% (246 of 269) of the KTs were incorrectly coded as intrahepatic CCs, resulting in an overestimation of intrahepatic CC incidence by 13% and underestimation of extrahepatic CC incidence by 15% [6]. This coding error also partly explains the rise in the incidence of intrahepatic CC in the United States over the last decade and a decrease in the incidence of extrahepatic CC [6]. Additionally, this reporting error of KT in the SEER cancer registries makes it impossible to define KT incidence precisely on a population-based level. The current study examined a large cohort of KT patients from the SEER database in an effort to precisely identify the demographic, clinical, and treatment strategies in the light of updated version 3 of ICD-O (ICD-O-3), which may impact the clinical outcomes in the current KT cohort.

This work has been presented as a poster at the 16th World Congress of Endoscopic Surgery (https://link.springer.com/article/10.1007/s00464-018-6121-4?code=74ed6187-bb4a-4f4e-aa9b-71da56ed25c1&error=cookies_not_supported).

## Materials and methods

Data for the current study were extracted from the SEER database provided by the National Cancer Institute between 2001 and 2012. SEER Stat software version 8.3.4 (National Cancer Institute, Bethesda, MD, USA) was used to extract data from 18 SEER registries (Alaska Native Tumor Registry, Arizona Indians, Cherokee Nation, Connecticut, Detroit, Georgia Center for Cancer Statistics, Greater Bay Area Cancer Registry, Greater California, Hawaii, Iowa, Kentucky, Los Angeles, Louisiana, New Jersey, New Mexico, Seattle-Puget Sound, and Utah). A total of 1,144 patients with histologically confirmed KT were identified, and their data were exported to IBM SPSS Version 20.2 (IBM Corp., Armonk, NY, USA).

A total of 1,144 patients with a primary diagnosis of KT were identified to form the final study cohort using the SEER International Classification of Disease for Oncology (ICD-O-3) codes 9508/3. Demographic and clinical data extracted included age, sex, race, tumor stage, tumor size, primary tumor site, and type of treatment received (surgery, radiation, both, or unknown/no therapy). The term “no treatment” refers to the lack of reported treatment. Patients with in situ cancers, those with a nonspecific site of tumor origin, and those in whom histologic confirmation of their cancer was not available were excluded from the final study cohort.

The endpoints examined included overall survival and cancer-specific mortality. Categorical variables were compared using the chi-square test, and continuous variables were compared using Student’s t-test and analysis of variance. Multivariable analysis using the “backward Wald” method was performed to calculate odds ratios (ORs) and determine the independent factors affecting survival. Missing and unknown data were excluded from multivariate analysis. The Kaplan-Meier analysis was used to compare the long-term actuarial survival between the groups. Statistical significance was set at p<0.05.

## Results

Demographic data

A total of 1,144 KT cases were reported in the SEER database over the 11-year study period (2001-2012). The mean age at diagnosis for KT was 73±13 years compared to all other CCs of the biliary tree (69±13 years, p<0.001; Table 1). There were 627 male patients (N=627 [54.8%], p=0.001) and 517 female patients (N=517 [45.2%], p=0.001), with a male-to-female ratio of 1.2:1. The majority of KT cases occurred among Caucasians (N=795[ 69.5%], p=0.016), followed by Hispanics (N=134 [11.7%], p=0.016), Asian (N=129 [11.3%], p=0.016), African-Americans (N=76 [6.6%], p=0.016), and others (N=10 [0.9%], p=0.016) (Table 1).

**Table 1:** Demographic profiles of 1,144 patients with KT from the SEER database (2001-2012) KT, Klatskin tumor; SEER, Surveillance, Epidemiology, and End Result

		Overall	Cholangiocarcinoma	Klatskin tumor	p-Values
N (%)		12,234	11,090	1,144	
Age (years)		70±13	69±13	73±13	<0.001
Sex, N (%)	Female	6,083 (49.7)	5,566 (50.2)	517 (45.2)	0.001
	Male	6,151 (50.3)	5,524 (49.8)	627 (54.8)	0.016
Race, N (%)	Caucasians	7,933 (64.8)	7,138(64.4)	795 (69.5)	<0.001
	African American	922 (7.5)	846(7.6)	76 (6.6)	
	Hispanic	1,695 (13.9)	1,561(14.1)	134 (11.7)	
	Asian	1,548 (12.7)	1,419(12.8)	129 (11.3)	

Tumor characteristics

The most common primary site for KT was the extrahepatic ducts (N=612[ 53.5%], p<0.001), followed by the intrahepatic duct (N=491 [42.9%], p<0.001), biliary tract, not otherwise specified (n=27; 2.4%, p<0.001), overlapping lesions (N=8 [0.7%], p<0.001), and gallbladder (N=6 [0.5%], p<0.001) (Table 2).

**Table 2:** Tumor characteristics of 1,144 patients with KT from the SEER database (2001-2012) KT, Klatskin tumor; NOS, not otherwise specified; SEER, Surveillance, Epidemiology, and End Result

		Overall	Cholangiocarcinoma	Klatskin tumor	p-Values
Primary site, N (%)	Intrahepatic duct	6,117 (50.0)	5,626 (50.7)	491 (42.9)	<0.001
	Gallbladder	529 (4.3)	523 (4.7)	6 (0.5)	
	Extrahepatic duct	4,883 (39.9)	4,271 (38.5)	612 (53.5)	
	Ampulla of Vater	51 (0.4)	51 (0.5)	0 (0)	
	Overlapping lesion	59 (0.5)	51 (0.5)	8 (0.7)	
	Biliary tract, NOS	595 (4.9)	568 (5.1)	27 (2.4)	
Grade, N (%)	Well differentiated	470 (308)	427 (3.9)	43 (3.75)	<0.001
	Moderately differentiated	1,633 (13.3)	1,561 (14.1)	72 (6.3)	
	Poorly differentiated	1,507 (12.3)	1,441 (13.0)	66 (5.8)	
	Undifferentiated	75 (0.6)	71 (0.6)	4 (0.3)	
	Unknown	8,549 (69.9)	7,590 (68.4)	959 (83.8)	
SEER stage, N (%)	Localized	2,447 (20.0)	2,119 (19.1)	328 (28.7)	<0.001
	Regional	3,413 (27.9)	3,068 (27.7)	345 (30.2)	
	Distant	3,897 (31.9)	3,722 (33.6)	175 (15.3)	
	Unstaged	2,477 (20.2)	2,181 (19.7)	296 (25.9)	
Lymph node, N (%)	Unknown	9,808 (84.6)	8,899 (84.4)	909 (86.9)	0.056
	N0	919 (7.9)	842 (8)	77 (7.4)	
	N+	865 (7.5)	805 (7.6)	60 (5.7)	
Tumor size, N (%)	Unknown	7,083 (61.1)	6,384 (60.5)	699 (66.8)	<0.001
	Microscopic	5 (0)	5 (0)	0 (0)	
	<2 cm	818 (7.1)	718 (6.8)	100 (9.6)	
	2-4 cm	1,387 (12)	1,243 (11.8)	144 (13.8)	
	>4 cm	2,299 (19.8)	2,196 (20.8)	103 (9.8)	

Overall, 673 KT cases (N=673 [58.9%], p<0.001) were locoregional at presentation, 175 cases (N=175 [15.3%], p<0.001) had distant metastasis, and 296 cases (N=296 [25.9%], p<0.001) were not staged. Histologically, moderately differentiated tumors were the most common (38.9%), followed by poorly differentiated (35.7%), well-differentiated (23.3%), and undifferentiated tumors (2.2%) (p<0.001). Most tumors in the KT group were 2-4 cm in size (N=144 [41.5%], p≤0.001), while fewer were >4 cm (N=103 [29.7%], p≤0.001) and <2 cm (N=100 [28.8%], p≤0.001). The majority of KT cases had no information related to lymph node involvement (N=909 [86.9%], p=0.0560, while 77 (7.4%, p=0.056) were N0 at presentation and 60 (5.7%, p=0.056) were N+ at presentation (Table 2).

Treatment

The majority of KT patients (N=835 [73%], p<0.001) were offered no treatment. In KT patients who were offered treatment, the most frequent modality was radiation (N=148 [52.7%], p<0. 001), followed by surgery (N=79 [28.1%], p≤0. 001), and both surgery and radiation (N=54 [19.2%], p<0.001) (Table 3).

**Table 3:** Treatment and survival outcomes of 1,144 patients with KT from the SEER database (2001-2012) KT, Klatskin tumor; SEER, Surveillance, Epidemiology, and End Result

		Overall	Cholangiocarcinoma	Klatskin tumor	p-Values
Treatment, N (%)	No treatment	8,403 (68.7)	7,568 (68.2)	835 (73.0)	<0.001
	Surgery only	1,628 (13.3)	1,549 (14.0)	79 (6.9)	
	Radiation only	1,018 (8.3)	870 (7.8)	148 (12.9)	
	Both	641 (5.2)	587 (5.3)	54 (4.7)	
	Unknown	544 (4.4)	516 (4.7)	28 (2.4)	
Survival by treatment, year±SD (%)	None	0.57±1.04	0.57±1.03	1.72±2.61	0.807
	Surgery alone	1.87±2.20	1.87±2.18	1.72±2.61	0.047
	Radiation alone	1.00±1.17	0.99±1.15	1.08±1.26	0.375
	Both	2.09±2.12	2.10±2.10	2.01±2.29	0.575
Mean survival (years)		0.87±1.45	0.88±1.45	0.83±1.48	0.007
Overall cumulative survival, N (%)	1-year	3,220 (26.3)	2,941 (26.5)	279 (24.4)	
	2-year	1,407 (11.5)	1,281 (11.6)	126 (11.0)	
	5-year	333 (2.7)	301 (2.7)	32 (2.8)	

Outcomes

**Figure 1:**
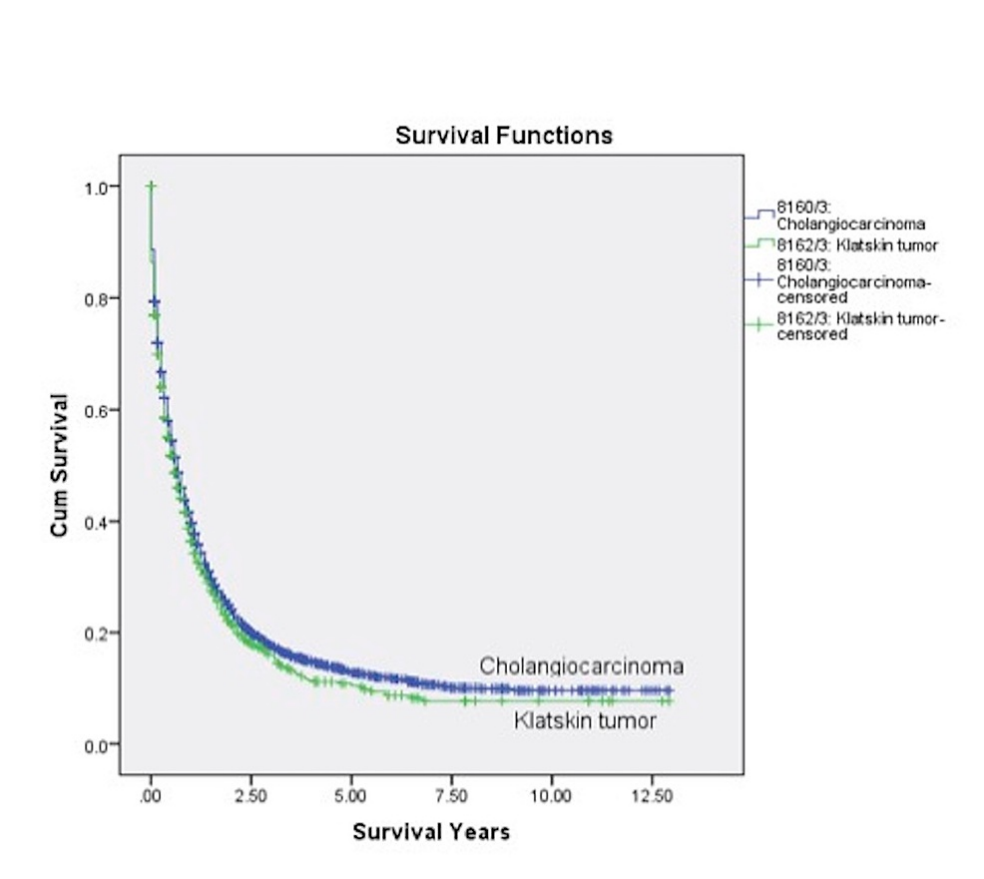
The Kaplan–Meier curves illustrating actuarial survival for patients with KT from the SEER database (2001-2012) KT, Klatskin tumor; SEER, Surveillance, Epidemiology, and End Result

The longest survival was seen among KT patients receiving both surgery and radiation (2.0±2.3 years), followed by surgery alone (1.7±2.6 years), those receiving neither surgery nor radiation (1.7±2.6 years), and finally those receiving radiation alone (1.0±1.3 years), but it was not statistically significant. Additionally, the mean survival time for those KT patients treated with surgery was inferior to all CCs of the biliary tree (1.72±2.61 vs. 1.87±2.18 years; p=0.047). The overall cumulative survival for KT at one year was 24.4% (N=279), at two years was 11% (N=126), and at five years was 2.8% (N=32) (Figure 1, Table 3).

Multivariable analysis

Multivariate analysis identified regional metastasis (OR=2.8; 95% CI=2.6-3.0), distant metastasis (OR=2.1; 95% CI=1.9-2.4), lymph node positivity (OR=1.6; 95% CI=1.4-1.8), Caucasian race (OR=2.0; 95% CI=1.8-2.2), and male sex (OR=1.2; 95% CI=1.1-1.3) to be independently associated with increased mortality for KT (p<0.001) (Table 4).

**Table 4:** Multivariate analysis of factors influencing mortality in patients with Klatskin tumor

	Odds ratio (95% CI)	p-Value
Male gender	1.2 (1.1-1.3)	0.001
Caucasian race	2.0 (1.8-2.2)	0.001
Regional metastasis	2.8 (2.6-3.0)	0.001
Distant metastasis	2.1 (1.9-2.4)	0.001
Lymph node positivity	1.6 (1.4-1.8)	0.001

## Discussion

KT is an uncommon malignant tumor originating from the epithelium of the common hepatic duct or its first and second bifurcations [2]. Previous studies on CC have reported an increasing incidence of intrahepatic CC and a decreasing incidence of extrahepatic CC in the United States [7,6]. KTs are anatomically defined as extrahepatic CCs and could have affected these trends. In version 1 of ICD-O (1973-1991), KTs were not assigned a unique ICD-O code and were coded as either intrahepatic CC or extrahepatic CC [6]. In ICD-O version 2, KTs were assigned a unique histology code (8162/3), which also included intrahepatic CC. Thus, KTs may have been misclassified as intrahepatic CCs under these versions of ICD-O [6].

Based on the current study, KT is most prevalent among male Caucasians in the seventh decade of life, which is consistent with previous studies [8]. Also, KTs are most commonly located in extrahepatic ducts, exhibit a locoregional spread, and have a tumor size of 2-4 cm. Additionally, the current study also found that distant metastasis occurred in 15.3% of KT patients. A careful review of the literature demonstrates that tumor extension with lymph node metastasis and neural invasion is a characteristic feature of KT, with the incidence of nodal involvement in resectable tumors ranging from 30% to 50% [9-13]. To assess the status of the regional and para-aortic lymph nodes in KT, Kitagawa et al. in an institutional study of 110 patients found that pericholedochal nodes in the hepatoduodenal ligament were the most common sites of metastasis for KT [9].

Histologically, KTs are mostly moderately to well-differentiated biliary type adenocarcinomas, which is consistent with the findings of this study [14]. These tumors are characterized by abundant tubules and glands in a typical desmoplastic stroma along with a variable inflammatory response [14]. Furthermore, advances in radiological imaging have permitted better delineation and improved sensitivity in detecting KT lesions [15]. Traditionally, the initial radiographic assessment for KT has always been a transabdominal ultrasound, which is cost-effective and easily accessible but cannot determine the type of obstruction and extent of tumor involvement [15]. Computed tomography (CT) is the most frequently used imaging modality and demonstrates an acceptable accuracy (>80%) in assessing ductal, portal vein, and hepatic artery involvement; however, it cannot accurately determine lymph node involvement and underestimates peritoneal involvement [15,16].

Recently, magnetic resonance cholangiopancreatography (MRCP) has gained tremendous popularity among surgeons owing to its ability to precisely predict the resectability of KT (>80%) [16-18]. KT appears as a hypointense signal on T1-weighted images and with high signal intensity on T2 imaging [15,19]. In addition, the role of positron emission tomography PET/CT in evaluating the local resectability of KT remains unclear [20]. Currently, it may be useful when assessing metastatic disease but has no clear role in helping to evaluate issues of local resectability [15,19,20].

Traditionally, KT is treated with surgical resection alone. Although surgical resection with negative margins is the only hope for a cure, only a small subset of patients is amenable to surgery at the time of diagnosis [21,22]. Complete surgical resection is the most critical prognostic factor for survival; however, total or near-total resection is challenging because of the close anatomical relationship of the bile duct bifurcation with the portal vein bifurcation and hepatic arteries [5,22]. In the current study, most of the KT patients who were offered treatment received radiation alone as a primary treatment, followed by surgery, and the survival was poor (1.08±1.26 years).

The role of adjuvant or neoadjuvant radiation therapy in the management of KT has always been controversial owing to the lack of prospective randomized controlled trials [23]. Some retrospective studies have demonstrated the beneficial effects of radiation therapy in augmenting survival rates in patients with KT. In a recent study from Japan, Todoroki et al. examined 63 patients with stage IVa KT, of which 21 patients underwent resection only and 42 patients received either intraoperative radiation therapy (IORT), postoperative radiation therapy (PORT), or both [24]. The locoregional control rate was significantly greater in the adjuvant therapy group than in the resection alone group (79.2% vs. 31%). The actual five-year survival was also significantly improved in patients treated with resection + IORT + PORT (39.2%) than in those who received resection alone (13.5%) (p=0.0141) [24].

Furthermore, the role of chemotherapy in the management of KT is not well established. To the best of our knowledge, the most extensively investigated chemotherapeutic agent for KT management is 5-fluorouracil (5-FU) [25]. Several small studies have used single-agent systemic chemotherapy drugs including 5-FU, cisplatin, rifampicin, mitomycin C, paclitaxel, and gemcitabine [25]. These studies failed to establish an acceptable response rate and efficacy of the single-agent chemotherapeutic regimen for the management of KT [25].

Because of the poor response rates with single-agent chemotherapy therapy, several authors have used combination chemotherapy in an attempt to achieve better response rates and longer survival. A prospective randomized trial by the Eastern Cooperative Oncology Group (ECOG) led by Falkson et al. compared 34 patients with unresectable KT treated with either oral 5-FU or cyclonexyle-chloroethyl-nitrosourea (CCNU), demonstrating a partial response rate of only 9% [26].

Limitations

There are several limitations to this study that should be considered. First, the SEER database does not accurately code for all critical clinical factors such as socioeconomic status, geography, tumor depth, and method of diagnostic confirmation, which may have influenced survival. Second, information on diagnostic imaging and follow-up is lacking. Data on surgical and radiation therapy were available in the SEER database; however, data on chemotherapy received were not, which limited the ability of this study to evaluate the impact of adjuvant or neoadjuvant therapy. There may also be an element of selection bias since SEER registries are more likely to sample from urban areas than from rural areas. Despite these limitations, the SEER database has data obtained from 14% of the U.S. population, and these findings can be generalized to the overall population.

## Conclusions

KT is a rare and highly malignant tumor of the biliary tract that is associated with poor survival. A considerable error in reporting KT was observed in the SEER ICD-O-2 classification system using a histological code for KT, which also included intrahepatic CC. To our knowledge, the current study represents the largest KT cohort according to the updated ICD-O-3 classification in the SEER database, establishing a more precise reporting of the demographics, management, and clinical outcomes. KT is more common among Caucasian males in the seventh decade of life and tends to occur in the extrahepatic ducts with locoregional presentation and size of 2-4 cm in size, with up to 15.3% of the patients developing distant metastasis. Although surgery remains the primary method of treatment for KT, radiation therapy in some studies has emerged as a promising adjunct for treatment, increasing the overall survival. Future studies optimizing the dosage of radiation regimens to establish the relationship between the multimodal approach for the treatment and its impact on survival are needed. All KT patients should be enrolled in clinical trials or registries to allow for more defined multimodality management to optimize clinical outcomes for these patients.
